# Vitamin D deficiency causes inward hypertrophic remodeling and alters vascular reactivity of rat cerebral arterioles

**DOI:** 10.1371/journal.pone.0192480

**Published:** 2018-02-06

**Authors:** Éva Pál, Leila Hadjadj, Zoltán Fontányi, Anna Monori-Kiss, Zsuzsanna Mezei, Norbert Lippai, Attila Magyar, Andrea Heinzlmann, Gellért Karvaly, Emil Monos, György Nádasy, Zoltán Benyó, Szabolcs Várbíró

**Affiliations:** 1 Institute of Clinical Experimental Research, Semmelweis University, Budapest, Hungary; 2 2^nd^ Department of Obstetrics and Gynecology, Semmelweis University, Budapest, Hungary; 3 Department of Physiology, Semmelweis University, Budapest, Hungary; 4 Department of Pathology, Jász-Nagykun-Szolnok County Hetényi Géza Hospital, Szolnok, Hungary; 5 Department of Anatomy, Histology and Embryology, Semmelweis University, Budapest, Hungary; 6 Department of Laboratory Medicine, Semmelweis University, Budapest, Hungary; 7 Bionics Innovation Center, Budapest, Hungary; "INSERM", FRANCE

## Abstract

**Background and purpose:**

Vitamin D deficiency (VDD) is a global health problem, which can lead to several pathophysiological consequences including cardiovascular diseases. Its impact on the cerebrovascular system is not well understood. The goal of the present work was to examine the effects of VDD on the morphological, biomechanical and functional properties of cerebral arterioles.

**Methods:**

Four-week-old male Wistar rats (n = 11 per group) were either fed with vitamin D deficient diet or received conventional rat chow with per os vitamin D supplementation. Cardiovascular parameters and hormone levels (testosterone, androstenedione, progesterone and 25-hydroxyvitamin D) were measured during the study. After 8 weeks of treatment anterior cerebral artery segments were prepared and their morphological, biomechanical and functional properties were examined using pressure microangiometry. Resorcin-fuchsin and smooth muscle actin staining were used to detect elastic fiber density and smooth muscle cell counts in the vessel wall, respectively. Sections were immunostained for eNOS and COX-2 as well.

**Results:**

VDD markedly increased the wall thickness, the wall-to-lumen ratio and the wall cross-sectional area of arterioles as well as the number of smooth muscle cells in the tunica media. As a consequence, tangential wall stress was significantly lower in the VDD group. In addition, VDD increased the myogenic as well as the uridine 5’-triphosphate-induced tone and impaired bradykinin-induced relaxation. Decreased eNOS and increased COX-2 expression were also observed in the endothelium of VDD animals.

**Conclusions:**

VDD causes inward hypertrophic remodeling due to vascular smooth muscle cell proliferation and enhances the vessel tone probably because of increased vasoconstrictor prostanoid levels in young adult rats. In addition, the decreased eNOS expression results in endothelial dysfunction. These morphological and functional alterations can potentially compromise the cerebral circulation and lead to cerebrovascular disorders in VDD.

## Introduction

Vitamin D deficiency (VDD) or insufficiency affects 1 billion people from all age groups worldwide. In addition to its well-characterized roles in calcium and phosphate homeostasis as well as in bone metabolism, 1,25-dihydroxyvitamin D—the active metabolite of vitamin D (VitD)—has numerous biological actions [1]. Besides interacting with the intracellular VitD receptor and regulating the expression of up to 200 genes, it mediates non-genomic actions as well [2]. VDD is associated with an increased risk of malignant tumor formation, autoimmune and infectious diseases as well as depression [1]. Diabetes mellitus and metabolic syndrome are also linked to VDD, as 1,25-dihydroxyvitamin D improves β-cell function and insulin sensitivity [3]. There is a growing body of evidence linking VDD to cardiovascular diseases including hypertension, atherosclerosis and coronary artery disease. Furthermore, a direct impact of VDD on endothelial dysfunction, arterial stiffness and vascular inflammation was also reported [2, 4, 5].

The effects of VDD on the cerebrovascular system are as yet less understood, although several studies highlight the importance of sufficient VitD status in cerebrovascular health. Low concentrations of VitD are associated with an increased risk of cerebrovascular diseases including ischemic stroke [6–9] and with poor poststroke outcome [10]. In addition, VDD is linked to chronic brain injury associated with cerebral small vessel disease [11]. The effect of VitD status on stroke severity was confirmed in rodent models as well: the infarction volume was larger and more severe poststroke behaviour impairment was observed in VitD-deficient rats as compared to VitD-sufficient ones [12].

In the present study, we hypothesized that the aforementioned adverse effects of VDD in stroke are related—at least in part—to VDD-induced alterations in cerebral arterioles. Therefore, we aimed to analyze the changes in the morphological, biomechanical and functional properties of the anterior cerebral artery (ACA) in a rodent model of VDD.

## Materials and methods

### Animals

All procedures conformed to the Guide for the Care and Use of Laboratory Animals published by the US National Institutes of Health (8th edition, 2011) and the EU-conform Hungarian Law on Animal Care (XXVIII/1998). The Institutional Animal Care and Use Committee of Semmelweis University approved the study protocol (IRB: 8/2014, PEI/001/1548-3/2014). All surgery was performed under sodium pentobarbital anesthesia, and all efforts were made to minimize suffering. Twenty-two 4-week-old male Wistar rats were involved in the experiments. Eleven of them were fed with VitD-deficient diet (VitD content less than 5 IU/kg) for eight weeks (VDD group). Control animals received conventional rat chow (containing 1500 IU/kg VitD) with per os VitD (Vigantol, 20.000 IU/mL cholecalciferol) supplementation (at the second week of the study 500 IU/100 g body weight as loading dose, then 140 IU/100 g b. w. weekly from week 4 to 7 as maintenance dose). Taken together, the daily VitD intake in the Control group was approximately 300 IU/100 g b. w. providing optimal VitD supply. Serum 25-hydroxyvitamin D, testosterone, androstenedione and progesterone levels were measured from blood samples taken at weeks 4 and 8. At week 6 a glucose tolerance test was performed: fasting as well as 60-minute and 120-minute postload plasma levels were measured before and after oral administration of 2 g/kg b. w. 30% glucose solution.

After 8 weeks, animals were sacrificed as follows. First, blood pressure was measured by cannulation of the carotid artery under general surgical anesthesia (pentobarbital, 45 mg/kg b. w., i. p.). After perfusion *via* the carotid artery with heparinized Krebs-Ringer solution and decapitation under anesthesia, the brain was removed and anterior cerebral artery (ACA) segments were prepared under a stereomicroscope (Wild M3Z, Heerbrugg, Switzerland). Heart and testes were also removed for weight measurement.

### Pressure microangiometry

An approximately 2-mm-long segment of the ACA was immersed into an organ chamber (Experimetria Ltd., Budapest, Hungary) filled with normal Krebs-Ringer solution, cannulated at both ends with microcannulas, and extended to its *in vivo* length. The chamber was placed on the stage of an inverted microscope (Leica, Wetzlar, Germany). Pressure-servo pumps (Living Systems, Burlington, VT, USA)–with the belonging pressure transducer (Living Systems, Burlington, VT, USA), which had been calibrated with a mercurial manometer—were connected to both cannulas and the arteries were pressurized to maintain 50 mmHg intraluminal pressure. The cerebral arterioles were allowed to equilibrate for 30 min at this pressure in normal Krebs-Ringer solution bubbled with a gas mixture containing 5% CO_2_, 20% O_2_ and 75% N_2_, and the temperature was kept at 37°C during the entire measurement. After equilibration, 10^−4^ mol/L UTP was added to the chamber and after a 5-min incubation the pressure was increased to 150 mmHg, then decreased to 0 mmHg; this challenge was then repeated once more. Thereafter, the pressure was increased from 0 to 150 mmHg in 10 mmHg steps. All these procedures were performed rapidly—within 5 minutes—to avoid activation of the myogenic response. After the arterioles were allowed to equilibrate at 50 mmHg intraluminal pressure for 10 min, 10^−6^ mol/L bradykinin was added to the vessel chamber and incubated for 5 min at 50 mmHg intraluminal pressure in order to evaluate endothelium-dependent vasorelaxation [13]. Finally, the passive diameter of vessels was measured: the organ chamber was filled with Ca^2+^-free Krebs solution and after 20 min the pressure-diameter relationship was determined as described above. Pictures were taken during the experiment by a digital camera (Leica DFC 320) connected to the microscope. The outer and inner diameters of the vessels were measured by ImageJ image analysis software (Image J 1.5 NIH, USA). For the calibration, a micrometer etalon (Wild, Heerbrugg, Switzerland) was used.

### Calculations

Wall thickness was expressed as the difference between the outer and inner radii: *h = Ro-Ri*, where *h* is wall thickness, *Ro* is the outer radius and *Ri* is the inner radius. The wall cross-sectional area (*A*_*w*_) was computed according to the following equation: *A*_*w*_ = *(Ro*^*2*^*-Ri*^*2*^)*π. Tangential wall stress was computed according to the Laplace-equation: *σ*_*tang*_ = (P*Ri)/h, where *σ*_*tang*_ is the tangential wall stress and *P* is the intraluminal pressure. The elastic modulus was computed as: E = (ΔP/ΔRo)*2*Ri^2^*Ro/(Ro^*2*^*-Ri*^*2*^*)*, where *E* is the incremental elastic modulus, *ΔP* is the change in intraluminal pressure and *ΔRo* is the outer radius change in response to *ΔP*.

Incremental distensibility was computed as D = ΔV/V*ΔP, where *D* is the incremental distensibility and *ΔV* is the change in lumen volume relative to the initial volume *V* in response to pressure change (*ΔP*). Myogenic tone was computed as M% = 100*(Ri_*Cf*_*-Ri*_*nKR*_*)/Ri*_*Cf*_, where *Ri*_*Cf*_ is the inner radius in Ca^2+^-free solution and *Ri*_*nKR*_ is the inner radius in normal Krebs-Ringer solution. UTP-induced contraction was expressed as a percentage of complete relaxation: *T*_*UTP*_% = 100*(Ri_*Cf*_-*Ri*_*UTP*_*)/Ri*_*Cf*_, where *Ri*_*UTP*_ is the inner radius after incubation with UTP. Bradykinin-induced relaxation was expressed as a percentage of UTP-induced precontraction: *T*_*BK*_% = 100*(Ri_*BK*_*-Ri*_*UTP*_*)/Ri*_*UTP*_, where *Ri*_*BK*_ is the inner radius after bradykinin was added to the chamber.

### Histology and immunohistochemistry

ACA segments were freshly fixed with formalin for histological examination and stained for elastic fibers with Weigert’s resorcin-fuchsin. Paraffin-embedded sections of ACA were immunostained with anti-COX-2 antibody (1:500, 1 h, 37°C) or with anti-eNOS antibody (1:200, 1 h, 37°C) after deparaffinization and antigen retrieval (0.1 mmol/L citrate buffer, pH 6, 30 min, 60°C). Endogenous peroxidase activity was quenched (with hydrogen-peroxide dissolved in distilled water) and blocked (2.5% normal horse serum). After secondary antibody (biotinylated horse anti-mouse antibody for COX-2 or biotinylated horse anti-rabbit antibody for eNOS) staining (30 min, RT) the binding sites of primary antibodies were visualized with DAB Substrate Kit (with 3,3’-diaminobenzidine; 6 min, RT). Sections were counterstained by hematoxylin. Artery segments were stained for smooth muscle actin (36 min, 37°C) using Ventana Benchmark Ultra System after deparaffinization and antigen retrieval (8 min, 97°C). UltraView Universal DAB Detection Kit was used for detecting primary antibodies. Data collections were made by microscope (Zeiss AxioImager.A1) coupled with video-camera (Zeiss AxioCAm MRc5 CCD). RGB pictures of resorcin-fuchsin-stained segments were analyzed with the Leica Qwin image analysis software. The green levels (green color was chosen after evaluation of color histograms) were measured starting at the luminal surface and going radially in an outward direction. In addition, the thicknesses of the intimal and medial layers of arteries and the intima/media ratio were determined. Pictures of smooth muscle actin, eNOS and COX-2 staining were analyzed with the ImageJ image analysis software using the „Color deconvolution” profile. Optical density of the endothelial layer after eNOS and COX-2 staining and the nucleus count of tunica media after demarcation with smooth muscle actin staining were determined.

### Statistical analysis

All data are presented as mean±SEM. For statistical analysis Student’s t test or two-way repeated measures ANOVA followed by Bonferroni post hoc test was used; p<0.05 was considered to be statistically significant. GraphPad Prism version 6.0 was used as statistical software.

### Materials

Vigantol 20.000 IU/mL was obtained from Merck Serono (Mumbai, India). Vitamin D deficient (EF R/M, E15312-24) and conventional rat chow (SM R/M, S8106-S011) were purchased from ssniff Spezialdiäten GmbH (Soest, Germany). Pentobarbital was obtained from Ceva-Phylaxia (Budapest, Hungary). The composition of normal Krebs-Ringer solution (in mmol/L) was: NaCl 119; KCl 4.7; NaH_2_PO_4_ 1.2; MgSO_4_ 1.17; NaHCO_3_ 24; CaCl_2_ 2.5; glucose 5.5 and EDTA 0.034. The composition of Ca^2+^-free Krebs solution (in mmol/L) was: NaCl 92; KCl 4.7; NaH_2_PO_4_ 1.18; MgCl_2_ 20; MgSO_4_ 1.17; NaHCO_3_ 24; glucose 5.5; EGTA 2 and EDTA 0.025. Uridine 5’-triphosphate (UTP) and bradykinin were purchased from Sigma-Aldrich (Darmstadt, Germany). Anti-eNOS [14] and anti-COX-2 [14] antibodies were obtained from Abcam (Cambridge, MA, USA). Anti-smooth muscle actin antibody (1A4) [15] and UltraView Universal DAB Detection Kit were obtained from Ventana Medical Systems, Inc. (Tucson, AZ, USA). Secondary antibodies and DAB Substrate Kit [14] were purchased from Vector Laboratories (Burlingame, CA, USA).

## Results

### Physiological parameters and serum levels of hormones, 25-hydroxyvitamin D and glucose

At the end of the experiments the measured physiological parameters of the rats (body weight, heart/body weight ratio, testis weight, mean arterial blood pressure, heart rate, as well as serum testosterone, androstenedione and progesterone levels) did not differ between the two groups (Table 1), indicating that the VitD-deficient diet, at least within 8 weeks, does not affect these parameters. To assess the efficacy of VitD deprivation, serum 25-hydroxyvitamin D levels were measured and found to be significantly lower in VDD as compared to control animals at the 8^th^ week of treatment (Table 1). In the glucose tolerance test performed at the 6^th^ week of treatment, blood glucose levels before and after oral glucose administration did not show any difference between the two groups (Table 1). These findings exclude the possibility that any morphological or functional changes of the ACA in the present study would be secondary consequences of VDD-induced hypertension, metabolic syndrome or altered androgenic hormone status.

**Table 1:** Physiological parameters and serum levels of hormones, 25-hydroxyvitamin D and glucose in the two experimental groups.

MEASURED PARAMETER	CONTROL n = 11	VDD n = 11
Body weight *(g)*	435.7±17.7	444.5±10.3
Heart/body weight	0.34±0.01	0.34±0.01
Testis weight *(g)*	3.74±0.16	3.71±0.33
Mean arterial blood pressure *(mmHg)*	131±4	134±4
Heart rate (*1/min*)	357±18	348±11
Serum testosterone *(ng/mL)*	6.56±0.84	5.94±0.91
Serum androstenedione *(ng/mL)*	0.57±0.14	0.54±0.12
Serum progesterone *(ng/mL)*	14.42±2.31	19.86±2.61
Serum 25-hydroxyvitamin D *(ng/mL)*	19.66±0.81	3.59±0.21 ***
Glucose (OGTT 0 min) (*mmol/L)*	6.24±0.46	5.49±0.20
Glucose (OGTT 60 min) (*mmol/L)*	7.61±0.33	7.59±0.37
Glucose (OGTT 120 min) (*mmol/L)*	5.51±0.39	5.41±0.31

The body and testis weight as well as the heart/body weight ratio of rats did not differ between the groups. VDD did not influence the mean arterial blood pressure, the heart rate or the serum hormone levels. Serum levels of glucose did not show any significant difference between the two groups during the oral glucose tolerance test (OGTT). The VitD-deficient diet induced significantly lower serum 25-hydroxyvitamin D level (*** p<0.0001). All parameters except blood glucose levels were measured at the 8^th^ week of treatment. OGGT was performed at week 6. Data are presented as mean±SEM.

*** p<0.0001

### Arterial geometry

**Fig 1:**
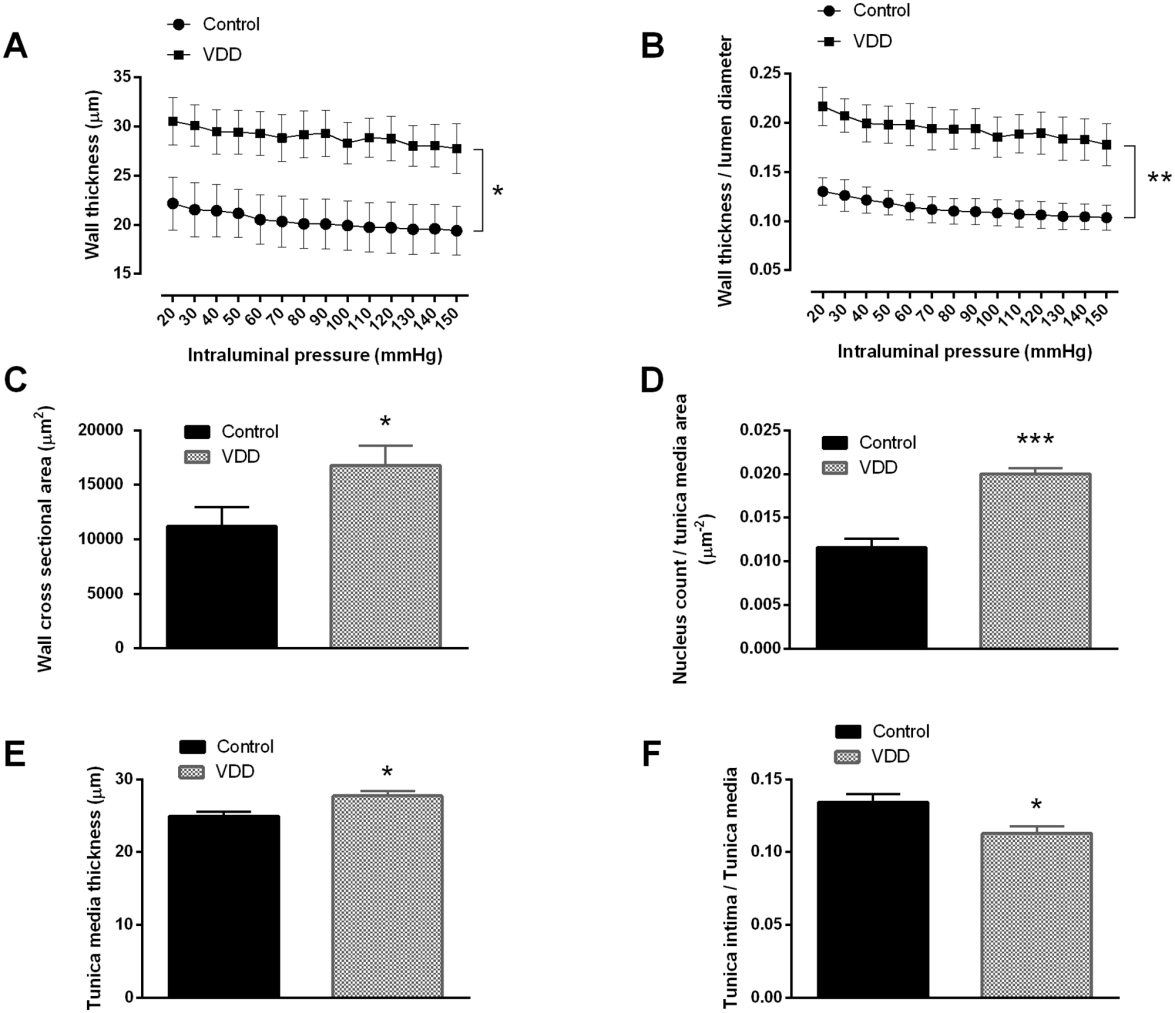
Cerebral artery geometry and nucleus count of the smooth muscle layer. (A) VDD significantly increased the wall thickness (* p<0.05, n = 10–11) as well as (B) the wall thickness / lumen diameter ratio (** p<0.01, n = 10–11). (C) The cross-sectional area of the vessel walls increased significantly in VDD as compared to the Control group (* p<0.05, n = 9–9) at 50 mmHg intraluminal pressure. All these parameters were measured under passive conditions. (D) Significantly more nuclei were detected in the smooth muscle layer of anterior cerebral arteries of VDD animals as compared to controls (*** p<0.0001, n = 4–6). (E) The thickness of the tunica media was increased in the VDD group as compared to the control animals (* p<0.05, n = 4–4). (F) In addition, VDD significantly decreased the intima / media ratio of cerebral arteries (* p<0.05, n = 4–4).

The artery segments were examined in Ca^2+^-free Krebs solution with pressure myograph to determine vessel geometry under passive conditions, as we hypothesized that a VitD-deficient diet could cause remodeling of cerebral arteries. In Ca^2+^-free Krebs solution the artery segments showed their fully relaxed inner radii, which decreased tendentiously in the VDD as compared to the Control group, but the difference did not reach the level of statistical significance (89.6±6.3 μm for Control, 78.4±5.6 μm for VDD, at 50 mmHg intraluminal pressure). However, wall thickness (Fig 1A) and wall thickness / lumen diameter ratio (Fig 1B) showed a significant increase in the VDD group under passive conditions. In addition, VitD-deficient feeding increased the wall cross-sectional area as compared to the Control group (Fig 1C), indicating the development of hypertrophic remodeling in the VDD group.

### Arterial elasticity

**Fig 2:**
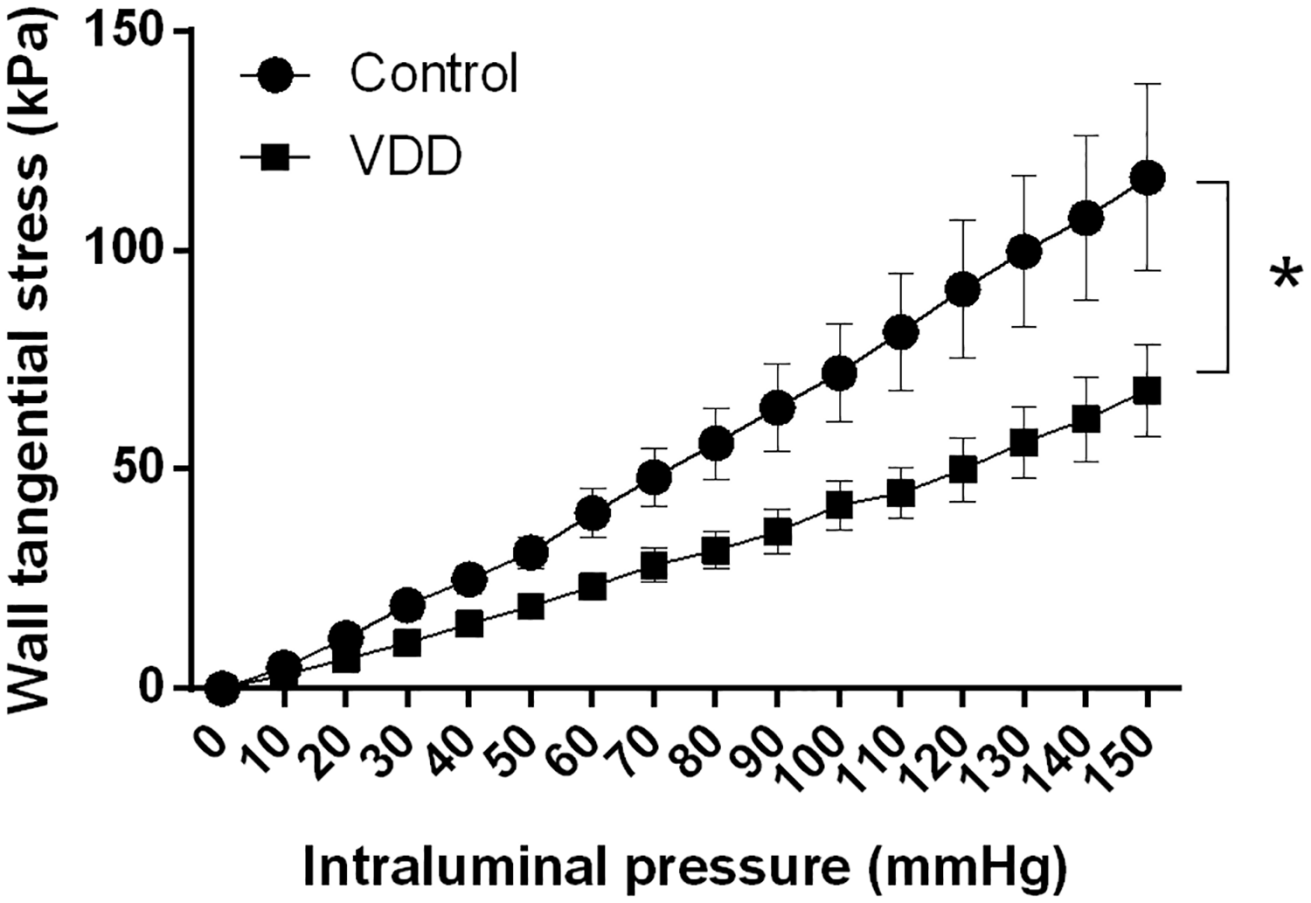
The tangential wall stress of the cerebral arteries in relaxed condition. VDD caused a decrease in the tangential stress of the vessel wall throughout the entire pressure range under passive conditions (* p<0.05, n = 10–11).

As previously mentioned, the wall thickness / lumen diameter ratio increased in the arterioles of the VDD group. In accordance with this finding, the tangential wall stress was significantly lower in the VDD group under passive conditions (Fig 2), as this biomechanical parameter is inversely proportional to the wall-to-lumen ratio. The incremental elastic modulus and the distensibility did not change significantly between the groups at 50 mmHg intraluminal pressure measured under passive conditions (elastic modulus: 2.89±0.27 log(kPa) and 2.65±0.19 log(kPa), distensibility: 1.41±0.12 log(Pa^-1^) and 1.18 ± 0.23 log(Pa^-1^) for the Control and VDD groups, respectively).

### Smooth muscle tone and endothelial reactivity

**Fig 3:**
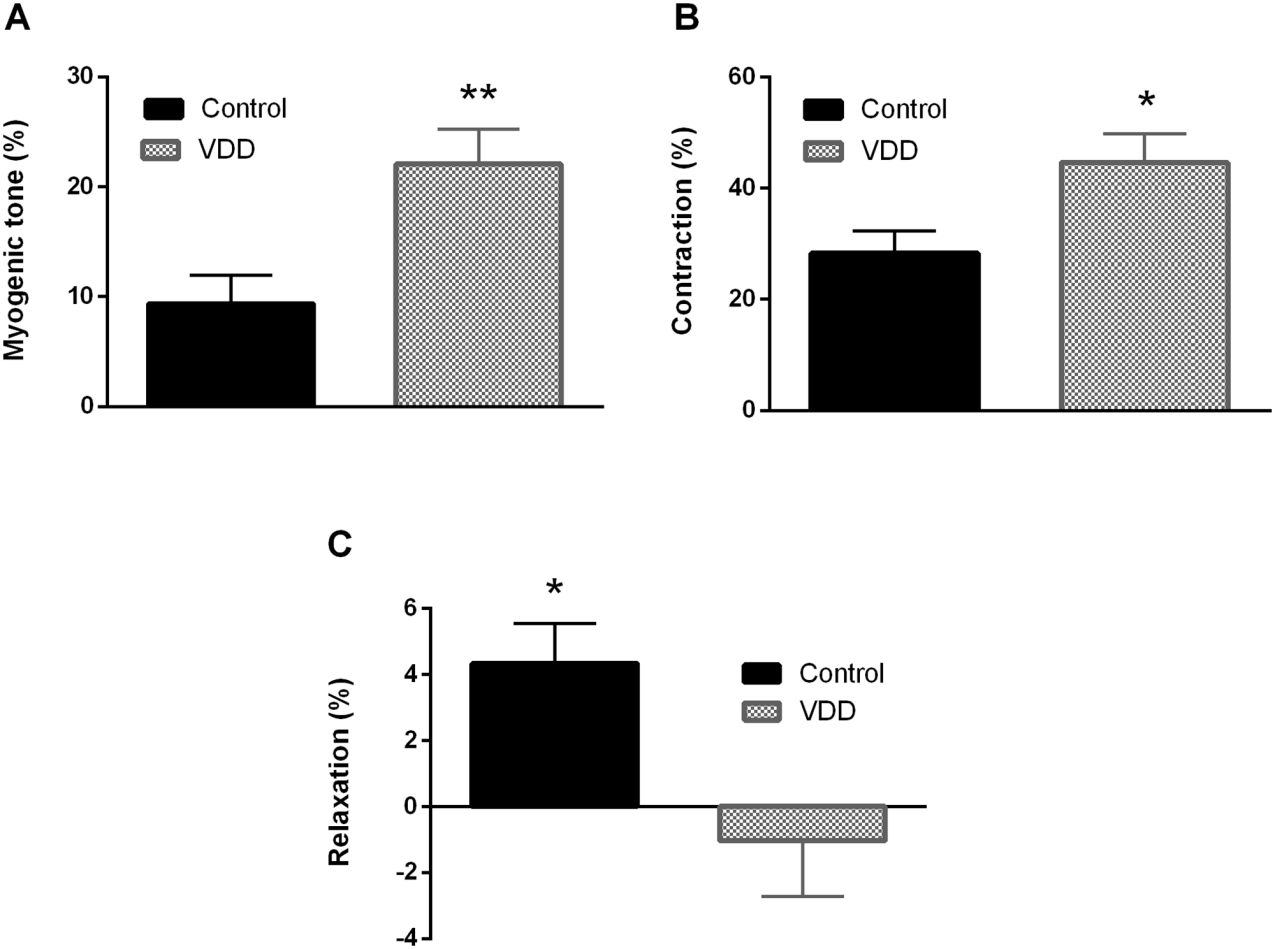
Alterations of vascular reactivity in VDD. VDD caused alterations in the smooth muscle tone and endothelium-dependent relaxation capacity of arterioles. (A) Anterior cerebral arteries possessed myogenic tone and this tone was greater in the VDD group measured at 50 mmHg intraluminal pressure (** p<0.01, n = 8–8). (B) In addition, UTP-induced contraction also increased in the VDD group at 50 mmHg intraluminal pressure (* p<0.05, n = 10–9). (C) Bradykinin induced endothelium-dependent relaxation after precontraction in the Control group, but did not relax the arterioles in the VDD group at 50 mmHg intraluminal pressure (* p<0.05, n = 9–9).

ACA possesses myogenic tone under resting conditions as well [16]. In our present experiment, the myogenic tone values of excised ACA segments were determined at 50 mmHg intraluminal pressure. Segments from VDD animals showed a two-fold increase in myogenic tone as compared to control ones, indicating that the 8-week VitD deprivation doubled the spontaneous tone of vessels (Fig 3A). Because UTP is a potent vasoconstrictor of cerebral arteries [17], the agonist-induced responsiveness with this agent was also tested at 50 mmHg intraluminal pressure. UTP caused potent constriction in both groups, but the induced tone was significantly greater in the VDD group (Fig 3B). To evaluate endothelial function, bradykinin was applied after precontraction with UTP. Whereas bradykinin induced slight vasodilatation in the Control group, it failed to relax the arteries from the VDD group (Fig 3C), indicating endothelial dysfunction in VitD-deficient animals.

### Histology and immunohistochemistry

**Fig 4:**
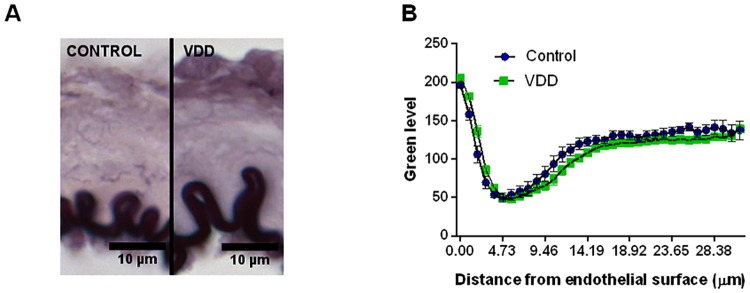
Elastic components of the vessel wall. (A) Representative images of cerebral arterioles stained with Weigert’s resorcin-fuchsin. (B) Elastic fiber density did not differ between the groups according to measurement of green color intensity as a function of distance from the luminal surface measured on resorcin-fuchsin-stained segments.

**Fig 5:**
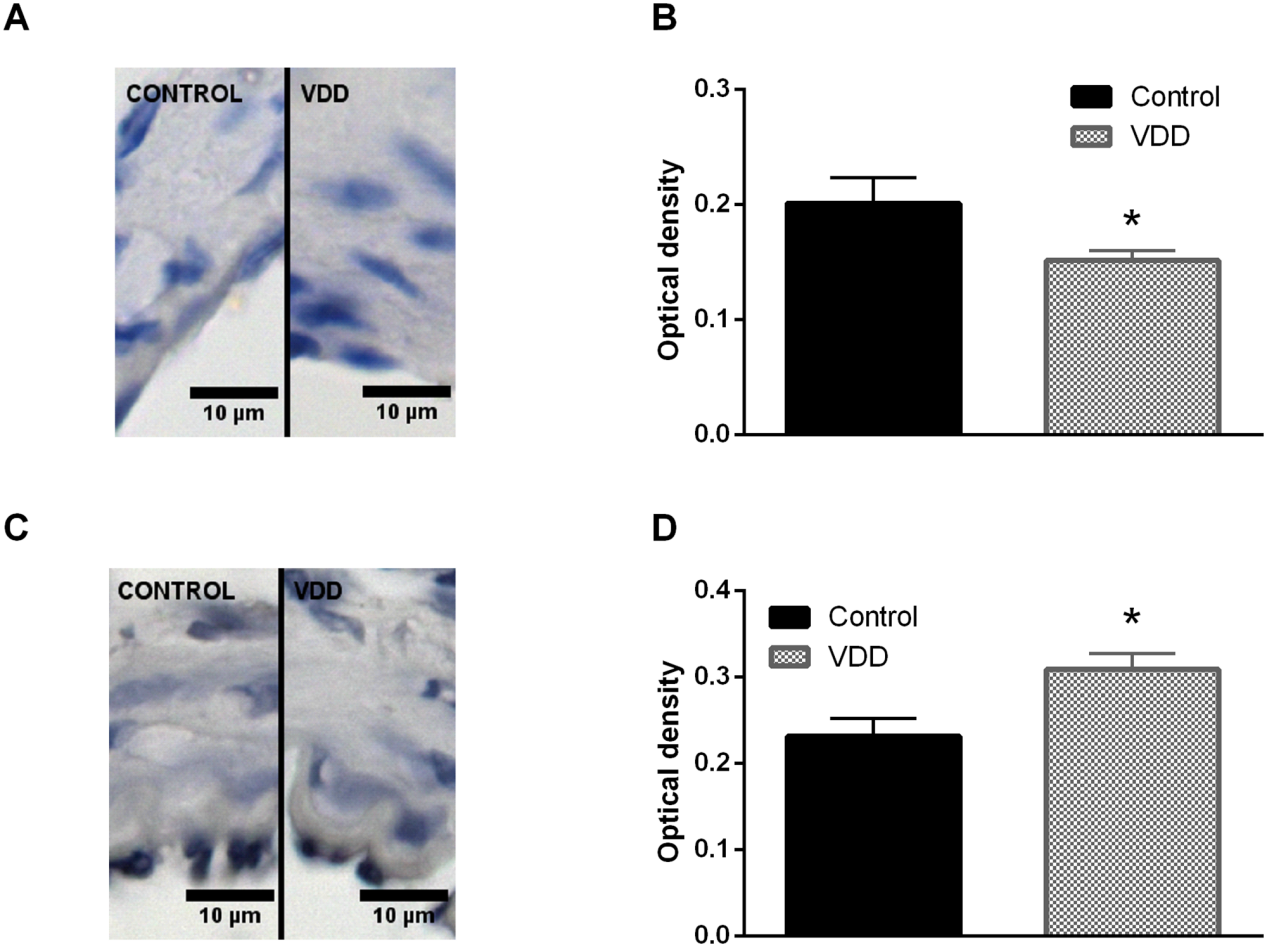
Evaluation of immunohistochemical staining of arterioles. (A) Representative immunohistochemical images of cerebral arterioles stained for eNOS. (B) VDD caused a decrease in the expression of eNOS in the endothelium of arterioles (* p<0.05, n = 4–6). (C) Representative immunohistochemical images of cerebral arterioles stained for COX-2. (D) The expression of COX-2 in the endothelial layer increased in VDD as compared to Control (* p<0.05, n = 4–4).

To gain further insight into the mechanism responsible for the above-mentioned observations, the density of the elastic components of the vessel wall was determined using resorcin-fuchsin staining, which did not show any difference between the groups based on the green color density measurements made in radial direction, outward from the luminal surface (Fig 4A and 4B). Furthermore, after immunohistological staining of smooth muscle actin the amount of nuclei was determined in the tunica media. The significantly increased nucleus count found in the smooth muscle layer of arteries from the VDD group (Fig 1D) indicated the presence of more vascular smooth muscle cells (VSMCs) in the vessel wall of VitD-deficient animals. Related to this alteration, increased thickness of the tunica media of arteries was observed in the VDD group (Fig 1E), however VDD did not affect the thickness of tunica intima (3.15±0.19 μm for Control, 3.15±0.24 μm for VDD). Therefore, VDD decreased the intima / media ratio of ACA (Fig 1F). To evaluate the possible role of NO and prostanoids in the effect of VDD, the expression of eNOS and COX-2 was determined by immunostaining. The optical density of endothelial eNOS staining was lower in the VDD group, indicating lower expression of eNOS (Fig 5A and 5B). In contrast, COX-2 expression was enhanced in the endothelial layer of arteries from the VDD group (Fig 5C and 5D).

## Discussion

To our knowledge, the present study provides the first report of the deleterious changes in geometry and reactivity of cerebral arteries due to VDD. These alterations were associated with hypertrophic remodeling, increased myogenic tone, endothelial dysfunction, increased COX-2 and decreased eNOS expression. Interestingly, marked changes of morphology and reactivity developed in healthy young adult animals within a relatively short period (8 weeks) of VDD indicating the importance of normal VitD status for the maintenance of cerebrovascular functions.

In humans, the cardiovascular consequences of VDD include hypertension, atherosclerosis, cardiac hypertrophy, cerebrovascular diseases, coronary artery disease, peripheral artery disease, dyslipidemia, insulin resistance and diabetes mellitus [2]; in addition, VDD during pregnancy leads to cardiomyopathy in infants [2, 18]. However, the associations, for instance, between VDD and insulin resistance [19, 20] or between VDD and hypertension [21] are not fully confirmed, especially in young healthy subjects [22–24]. The cardiovascular impacts of VitD are attributed to the modulation of immune, inflammatory and endothelial functions, furthermore to the regulation of VSMC proliferation and migration, renin expression, and extracellular matrix homeostasis [2].

In rodent models of VDD similar disorders of the cardiovascular system have been described as in humans, although their manifestation depends markedly on the experimental protocol applied. For instance, in a number of studies hypertension developed as a consequence of VDD, whereas the blood pressure remained unaltered in other studies (Table 2). According to the literature, VDD during the prenatal period leads to increased blood pressure in rodents’ offsprings [25–29] probably due to the upregulation of the renin-angiotensin system [18, 30]. Interestingly, VDD *in utero* contributes to the development of hypertension in later life even if the offsprings were fed with VitD sufficient chow after weaning [25, 26], which implies that VDD during pregnancy can severely impact the offspring’s long-term health and lead to vulnerability to cardiovascular disease in adult life [18, 31]. However, when VDD affects only the postnatal period of life, the observations are controversial both in growing and adult rodents; nevertheless longer exposure to VDD appears to increase the risk of manifestation of hypertension [25, 32–38]. Taken together, the development of hypertension appears to depend on the time of onset and the duration of VDD (Table 2). In our study, weanling rats were exposed to VDD for only 8 weeks, which explains the unaltered blood pressure levels. Although VDD is a complex disorder linked to several cardiovascular risk factors in the long run, we examined its short-term effects, and found altered cerebrovascular morphology and reactivity without the development of hypertension.

**Table 2:** Alterations of blood pressure in rodent models of VDD induced by VitD deficient feeding.

ONSET OF VDD	DURATION OF VDD (day)	SPECIES, STRAIN (M: mouse, R: rat)	INCREASE OF BLOOD PRESSURE (mmHg) [reference]
Prenatal	20	R, Sprague-Dawley	+15 [25]
Prenatal	30	M, Swiss Webster	+25 [26]
Prenatal	76	R, Sprague-Dawley	+10 [29]
Prenatal	76	M, C57BL/6 J	+ 20 [28]
Prenatal	200	M, BALB/c	+15 [27]
Juvenile	21	R, Sprague-Dawley	NO [32]
Juvenile	84	R, Wistar	+20 [33]
Juvenile	119	R, Lewis/SSN	NO [37]
Juvenile	196	R, Sprague-Dawley	+30 [38]
Adolescent	90	R, Wistar	+15 [35]
Pubescent	60	R, Wistar	NO [34]
Adult	28	R, Wistar	+10 [36]
Adult	70	R, Sprague-Dawley	NO [25]

Surprisingly, in spite of the well-preserved systemic cardiovascular and metabolic parameters, marked inward hypertrophic remodeling was observed in the cerebral arteries of VDD animals, similar to that observed in secondary hypertension [39]. In rats and humans suffering from secondary hypertension the increase in the wall-to-lumen ratio of small vessels is due to hypertrophic remodeling as a consequence of smooth muscle cell proliferation [39, 40]. In contrast, the hypertrophic remodeling observed in our present study develops at normal blood pressure and therefore is likely to be the direct effect of VDD on VSMCs. 1,25-dihydroxyvitamin D can modulate VSMC proliferation and migration [4]. In our study the increased cross-sectional area of the vessel wall appears to be the consequence of VSMC proliferation, because we observed increased number of smooth muscle cells in the tunica media of VitD-deficient arteries as compared to controls. On the contrary, we did not observe any changes in the tunica intima of arteries from the VDD group. The literature is controversial regarding the effects of VitD on VSMC proliferation and migration: some studies report enhanced migration and proliferation [41, 42], whereas others found VitD-induced inhibition of VSMC growth [43, 44]. VitD could inhibit VSMC proliferation *via* blunting c-myc RNA induction [42], up-regulating the negative modulators of cell proliferation including TGF-β [45] or decreasing cyclin-dependent kinase 2 activity [46]. The effect of VitD on VSMC definitely depends on the applied dose, and both insufficient and supraphysiological levels of VitD appear to lead to VSMC proliferation. In accordance, U-shaped association has been reported between VitD concentrations and cardiovascular diseases [4] [47]. Therefore, only physiological concentrations of VitD appear to be appropriate for maintaining normal VSMC function.

In hypertension, increased tangential wall stress facilitates wall thickening to compensate increased circumferential stress [48]. In our study, however, mean arterial blood pressure did not differ between the groups, thus the observed wall thickening resulted in decreased tangential wall stress according to the Laplace equation. The incremental elastic modulus and distensibility did not differ between the groups, indicating that elastic element density and arrangement were not influenced by VDD. This presumption was also confirmed by the unaltered elastic fiber density.

Cerebral arteries possess intrinsic myogenic tone [16], which can increase harmfully under pathophysiological conditions. In our study, arteries of VitD-deficient animals developed greater myogenic tone, which is similar to the observation of Tare et al., who reported a twofold enhancement of the myogenic tone of mesenteric arteries in male VitD-deficient rats as compared to VitD-sufficient ones [29]. In addition, UTP—a potent and partly thromboxane A_2_-mediated constrictor of cerebral arteries [49]—induced greater tone in the ACA of VDD animals. VitD is an important modulator of the prostanoid system, since it downregulates the expression of COX-2 [50], therefore VDD can lead to enhanced COX-2 expression, as we found in the endothelium of cerebral arteries. In addition, VitD suppresses the expression of TNF-α, NADPH oxidase and its subunits and also increases CuZn-SOD protein expression, therefore prevents inflammatory response and oxidative stress [51]. The increased level of reactive oxygen species (ROS) in VDD can in turn lead to enhanced vasoconstrictor response, as ROS inactivate prostacyclin synthase, shifting the prostanoid balance towards vasoconstriction [52]. Bradykinin relaxes cerebral arteries via the B_2_ receptor and NO release [13]; however, in the case of endothelial dysfunction bradykinin can cause endothelium-independent contractions [53]. VitD stimulates NO production through non-genomic eNOS activation via increase in eNOS phosphorylation [54] or due to an increase in eNOS mRNA and promoter activity depending on VitD receptor activation [55]. In our study, we observed decreased eNOS expression in the endothelium of VDD animals, which could contribute to the increased vessel tone and the impaired endothelium-dependent relaxation capacity. In the case of endothelial dysfunction, bradykinin-induced contractions might be mediated by COX-2 derived prostanoids and activation of thromboxane-prostanoid receptors [53]. The activation of thromboxane-prostanoid receptors could in turn impair the NO-mediated dilatation of vessels as well [56]. In addition, ROS can also contribute to the diminished endothelium-dependent vasodilatation in VDD due to eNOS uncoupling or inactivation of NO [57]. Enhanced vasoconstrictor prostanoid release and the impairment of the counterbalancing NO pathway could lead to increased myogenic tone and constrictor response as well as endothelial dysfunction in VDD.

VDD influences several pathways relevant to vascular physiology and pathophysiology, therefore it appears to be a significant risk factor for cardiovascular and cerebrovascular disease [2]. Since VDD is associated with dysfunction of endothelial and vascular smooth muscle cells, low VitD levels could predict proinflammatory and prothrombotic alterations, which might lead to atherosclerosis as well as increased thrombosis and arterial stiffness [2, 58]. VDD is not only a risk factor for coronary disease, acute myocardial infarction and stroke [2, 5, 59], but it also worsens the outcome when myocardial infarction and stroke are already present [10, 59]. In addition, low levels of VitD are associated with cerebral small vessel disease related vascular dementia [60, 61] and arterial stiffness associated cognitive impairment [62]. The observed alterations in cerebrovascular geometry and reactivity can be considered as prehypertensive changes [39]. Furthermore, the endothelial dysfunction of arteries may lead to the development of atherosclerosis in the long run, as the reduction of biosynthesis and enhanced inactivation of prostacyclin and NO are key components in the initiation of atherogenesis [63]. In addition, the increase in COX-2 expression as well as the proliferation of smooth muscle cells are also predictive of the development of atherosclerosis [63]. Therefore, long-term VDD with or without other disturbing pathophysiological conditions can in turn contribute to hypertension, atherosclerosis and a further increase in constrictor tone as well as to a more deleterious impairment of relaxation capacity, which can aggravate the risk of stroke events [64].

## Conclusions

The present study demonstrates the harmful effects of VDD on cerebral artery geometry and function in a rat model. We propose that VDD results in inward hypertrophic remodeling due to VSMC proliferation as well as in enhanced vessel tone due to increased vasoconstrictor prostanoid levels. In addition, impaired NO-mediated vasodilatation leads to endothelial dysfunction. Our results imply that a relatively short-term VDD in a relatively young age without any comorbidities can already induce marked morphological and functional alterations in the cerebral vasculature, which underlines the importance of sufficient VitD supply throughout the entire life in order to prevent stroke and other cerebrovascular diseases.
